# Supplementary material for: Mapping the increased minimum mortality temperatures in the context of global climate change
Source: Nat Commun. 2019 Oct 11;10:4640. doi: 10.1038/s41467-019-12663-y (PMC6789034; doi:10.1038/s41467-019-12663-y)
Supplement: Supplementary file 1 — Supplementary Information [file 41467_2019_12663_MOESM1_ESM.pdf]

Supplementary Information

Mapping the increased minimum mortality temperatures in the context of global climate change

Yin et al.

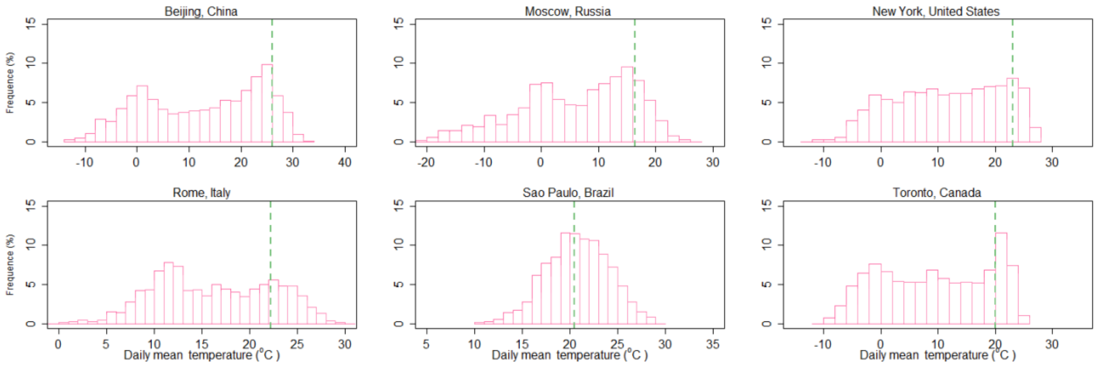

Supplementary Fig. 1 | The distributions of daily mean temperature (°C) in six representative cities during the study period. The green dashed lines show the MMTs observed in each city.

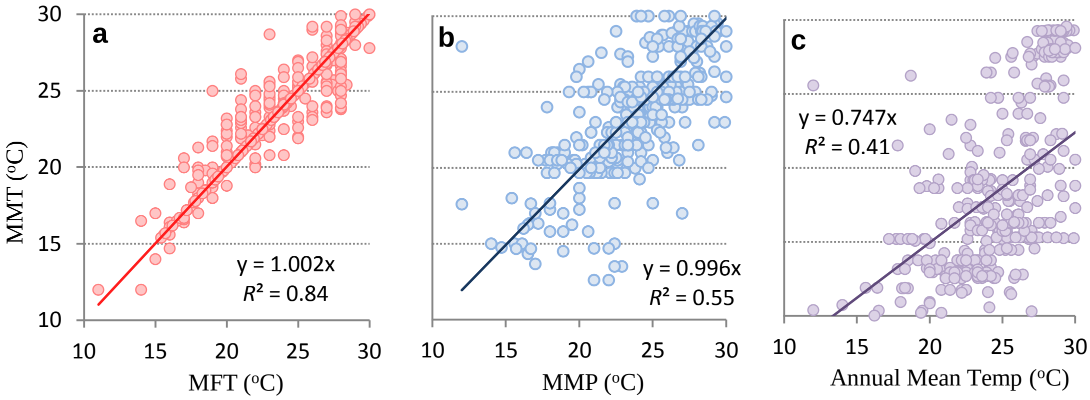

Supplementary Fig. 2 | Correlations of three temperature indicators and MMT. **a** for MFT. **b** for the 78th percentile temperature. **c** for annual mean temperature. The fitting lines and performances are shown in the figures. **a** MFT is proved to be the best indicator of MMT.

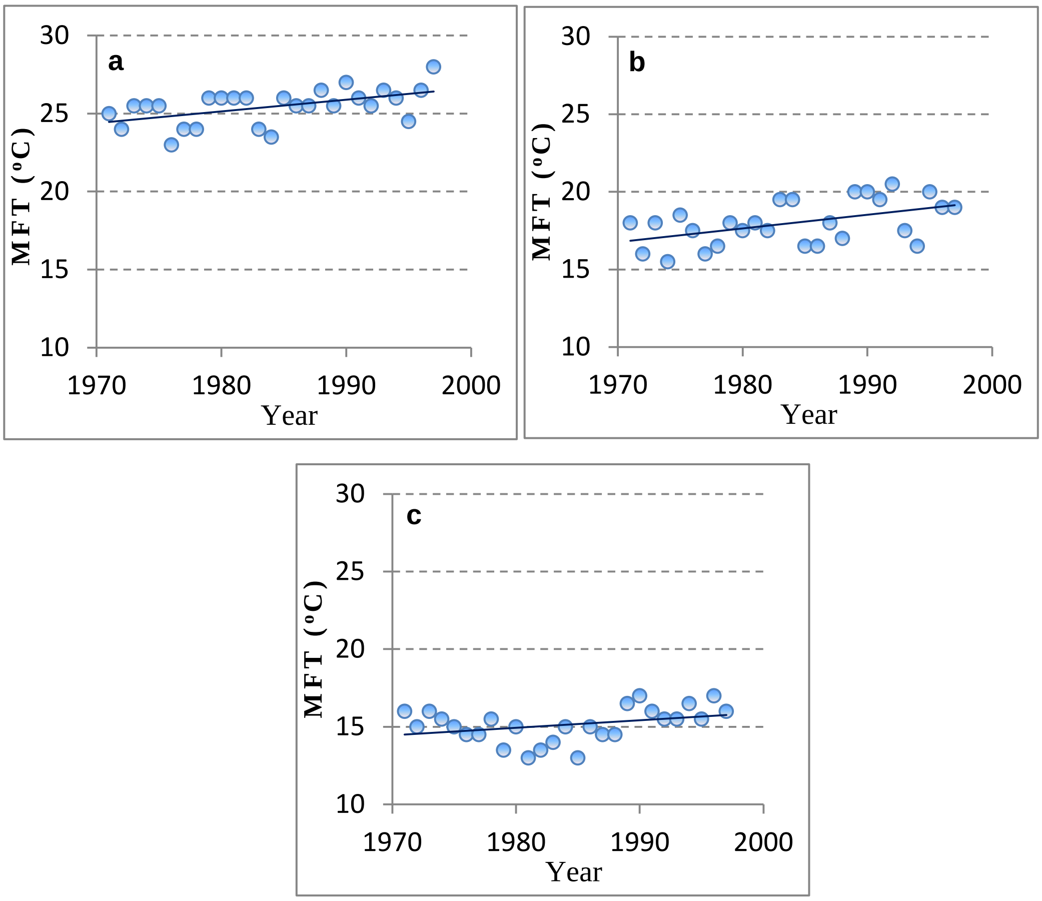

Supplementary Fig. 3 | The MFTs in three locations from 1971 to 1997. **a** for North Carolina. **b** for Southeast England. **c** for South Finland.

Parameters	Estimate	Std. error	*p*-value
MFT	0.70	0.03	< 2e-16 ***
Annual mean temperature	0.10	0.03	0.013*
78th percentile temperature	0.09	0.03	0.017*
Latitude	0.01	0.01	0.170
Annual temperature range	0.05	0.01	0.295
GDP/capita	0.00	0.00	0.379
Study year	0.05	0.02	0.299

Supplementary Table 1. Coefficients for Model 1 based on 420 MMTs.

Country	City	Longitude	Latitude	Study period
Russia	Moscow	37.4 °	55.5 °	2000 - 2006
Canada	Toronto	-79.3 °	43.0 °	1986 - 2009
Italy	Rome	12.5 °	41.8 °	1996 - 2007
USA	New York	-73.6 °	40.5 °	1985 - 2006
China	Beijing	116.5 °	39.9 °	2010 - 2012
Brazil	São Paulo	-47 °	-24.3 °	1997 - 2011

Supplementary Table 2. Descriptive statistics for 6 representative cities.
