# Supplementary material for: Mapping the increased minimum mortality temperatures in the context of global climate change
Source: Nat Commun. 2019 Oct 11;10:4640. doi: 10.1038/s41467-019-12663-y (PMC6789034; doi:10.1038/s41467-019-12663-y)
Supplement: Supplementary file 3 — Reporting Summary [file 41467_2019_12663_MOESM3_ESM.pdf]

nature research

Corresponding author(s): Jinfeng Wang (NCOMMS-19-00290-T)

Last updated by author(s): Sep 12, 2019

Reporting Summary

Nature Research wishes to improve the reproducibility of the work that we publish. This form provides structure for consistency and transparency in reporting. For further information on Nature Research policies, see Authors & Referees and the Editorial Policy Checklist.

Statistics

For all statistical analyses, confirm that the following items are present in the figure legend, table legend, main text, or Methods section.

n/a	Confirmed	
☐	☒	The exact sample size (*n*) for each experimental group/condition, given as a discrete number and unit of measurement
☐	☒	A statement on whether measurements were taken from distinct samples or whether the same sample was measured repeatedly
☐	☒	The statistical test(s) used AND whether they are one- or two-sided<br>*Only common tests should be described solely by name; describe more complex techniques in the Methods section.*
☐	☒	A description of all covariates tested
☒	☐	A description of any assumptions or corrections, such as tests of normality and adjustment for multiple comparisons
☐	☒	A full description of the statistical parameters including central tendency (e.g. means) or other basic estimates (e.g. regression coefficient) AND variation (e.g. standard deviation) or associated estimates of uncertainty (e.g. confidence intervals)
☐	☒	For null hypothesis testing, the test statistic (e.g. *F*, *t*, *r*) with confidence intervals, effect sizes, degrees of freedom and *P* value noted<br>*Give P values as exact values whenever suitable.*
☒	☐	For Bayesian analysis, information on the choice of priors and Markov chain Monte Carlo settings
☒	☐	For hierarchical and complex designs, identification of the appropriate level for tests and full reporting of outcomes
☐	☒	Estimates of effect sizes (e.g. Cohen's *d*, Pearson's *r*), indicating how they were calculated

Our web collection on statistics for biologists contains articles on many of the points above.

Software and code

Policy information about availability of computer code

Data collection	No software was used.
Data analysis	R software (Version X64 3.4.4)

For manuscripts utilizing custom algorithms or software that are central to the research but not yet described in published literature, software must be made available to editors/reviewers. We strongly encourage code deposition in a community repository (e.g. GitHub). See the Nature Research guidelines for submitting code & software for further information.

Data

Policy information about availability of data

All manuscripts must include a data availability statement. This statement should provide the following information, where applicable:

- Accession codes, unique identifiers, or web links for publicly available datasets
- A list of figures that have associated raw data
- A description of any restrictions on data availability

All the data used in our manuscript are obtained from open data. Data sources are available from the corresponding author upon request.

Field-specific reporting

Please select the one below that is the best fit for your research. If you are not sure, read the appropriate sections before making your selection.

☐ Life sciences ☐ Behavioural & social sciences ☒ Ecological, evolutionary & environmental sciences

For a reference copy of the document with all sections, see nature.com/documents/nr-reporting-summary-flat.pdf

Ecological, evolutionary & environmental sciences study design

All studies must disclose on these points even when the disclosure is negative.

Study description	By analysing the MMTs (minimum mortality temperature) in 420 locations across eight climate zones in the world, we found that although the MMT changes geographically, it is well represented by the local most frequent temperature.
Research sample	We reviewed a total of 210 papers, excluding the literatures from the repeated study areas, 16 papers of which investigated the relationships between temperature and human mortality counts for non-external causes using DLNM method. These studies involved 420 locations from 30 countries, covering six continents except Antarctica.
Sampling strategy	We applied a multiple linear regression model (MLR) to fit the MMT according to seven independent variables.
Data collection	Current temperature<br>A daily time series of the mean temperature of 420 locations during the study period was downloaded from the USA National Oceanic and Atmospheric Administration's (NOAA) website<br>Future temperature<br>The global projected daily mean temperatures from two representative concentration pathways (RCP4.5 and RCP8.5) under the five general circulation models (NorESM1-M, MIROC-ESM-CHEM, IPSL-CM5A-LR, HadGEM2-ES, and GFDL-ESM2M) during the 2010s and 2050s were obtained from the Inter-Sectoral Impact Model Inter-comparison Project (ISI-MIP).<br>Gross Domestic Product (GDP)<br>GDP/capita data was obtained from World Bank Open Data.
Timing and spatial scale	Time: From 1984 to 2014 years.<br>Space: Global
Data exclusions	No data was excluded.
Reproducibility	All attempts to repeat the experiment were successful.
Randomization	All the 420 samples data was obtained from the published studies, which covered a wide range of climates and populations with different demographic, socioeconomic, and infrastructural characteristics.
Blinding	All the data used in our manuscript are open data.

Did the study involve field work? ☐ Yes ☒ No

Reporting for specific materials, systems and methods

We require information from authors about some types of materials, experimental systems and methods used in many studies. Here, indicate whether each material, system or method listed is relevant to your study. If you are not sure if a list item applies to your research, read the appropriate section before selecting a response.

Materials & experimental systems

n/a	Involved in the study
☒	☐ Antibodies
☒	☐ Eukaryotic cell lines
☒	☐ Palaeontology
☒	☐ Animals and other organisms
☒	☐ Human research participants
☒	☐ Clinical data

Methods

n/a	Involved in the study
☒	☐ ChIP-seq
☒	☐ Flow cytometry
☒	☐ MRI-based neuroimaging
